# Responsive communication coaching for early childhood practitioners in underserved South African contexts: Clinical perspectives

**DOI:** 10.4102/sajcd.v66i1.608

**Published:** 2019-06-03

**Authors:** Shabnam S. Abdoola, Renata Mosca, Bhavani S. Pillay

**Affiliations:** 1 Department of Speech-Language Pathology and Audiology, University of Pretoria, Pretoria, South Africa

**Keywords:** Early childhood development, responsive communication coaching, inter-professional collaboration, participation, teamwork

## Abstract

Children spend longer hours with early childhood development (ECD) practitioners who are well-placed to facilitate critical early language development. ECD classrooms include a growing number of children at risk for communication delays. Greater collaboration between speech-language therapists (SLTs) and ECD practitioners is needed. Research highlights that responsivity coaching improves communication development. Therefore, responsive communication coaching was identified as a possible approach to early communication development within the classroom. This clinical perspective serves as a reflection on the programme by examining ECD practitioners’ perceptions thereof. Responsive communication coaching was identified as a means to improve practitioner–student collaboration within classrooms. This reflection aimed to describe ECD practitioners’ perceptions of responsive communication coaching implemented by student SLTs. Early childhood development practitioners were recruited from three sites in low to middle socio-economic settings, where most children were English additional language learners. Coaching was presented to 15 practitioners via 16 sessions conducted by student SLTs under supervision. Practitioners completed a custom-designed survey regarding their skill development and experiences of the coaching. All practitioners expressed benefit from coaching. Half of the practitioners (50%) rated coaching as very helpful, while 37% perceived it as helpful. The remaining practitioners (13%), based at the special needs preschool, perceived coaching as quite helpful. Thematic analysis identified the following benefits: enhanced interaction, improvements in children’s communication and the use of responsive communication strategies. Speech-language therapists need to collaborate with and support ECD practitioners in novel ways. The exploratory findings suggest that ECD practitioners benefit from SLT student-led responsive communication coaching sessions.

## Introduction

Undergraduate speech-language therapy (SLT) students at the University of Pretoria provide services in numerous early childhood development (ECD) settings as part of their clinical training. A traditional ‘pull-out’ approach to therapy was followed with minimal collaboration with ECD practitioners. Children showed benefits from the condition-centred intervention approach used; however, generalisation was limited because of poor ECD practitioner–student collaboration, and university examinations and holidays resulted in service delivery disruptions. Consequently, clinical supervisors recognised that the SLT services were inadequately aligned to the International Classification of Functioning and Disability – Children and Youth (ICF-CY) guidelines (World Health Organization [WHO], 2007), which encourage a holistic view of children within their environments. The clinical supervisors wanted to explore a service delivery method that would enable ECD practitioners and SLT students to shift their focus towards common child-centred goals, to optimise resources and facilitate early communication development within the classroom (Sheridan, Edwards, Marvin, & Knoche, 2009). Considering situational challenges and recent literature (Archibald, 2017; Glover, McCormack, & Smith-Tamaray, 2015), responsive communication coaching was identified as a possible new approach to early communication development.

Early communication skills lie at the core of ECD and later academic success (Rezzonico et al., 2015). The importance of ECD is well-recognised in literature and educational and health policies (South African Department of Education, 2008; Human Sciences Research Council, 2012). As many countries have made reception year (Grade R) compulsory, there is a greater focus on stimulation of foundational skills prior to formal schooling (Department of Education [DoE], 2001). As a result, families and communities have sought out ECD centres for their young children. Early childhood abilities are strong predictors of academic and social outcomes, and children who attend quality ECD programmes are more prepared for formal schooling (Duncan et al., 2007). However, children from low socio-economic settings (SES) are less likely to access ECD programmes than children from higher socio-economic contexts. Additionally, children in impoverished settings are more at risk for developmental delay, because of environmental and biological factors (Walker et al., 2011), and require greater support during early development (Samuels, Slemming, & Balton, 2012).

Globally, there is a disparity between learning settings available to young children, predominantly related to families’ socio-economic statuses (Alderman, 2011). Three hundred and eighty-five million children worldwide live in extreme poverty (Newhouse, Suarez-Becerra, & Evans, 2016). Nearly two-thirds of South African families experience stresses including reduced family resources and negative patterns of interactions, such as separation from caregivers because of migrant labour, which influence child development (Hall, Richter, Mokomane, & Lake, 2018; HSRC, 2012). Stressors can impact development over generations, maintaining the cycle of poverty and disability (HSRC, 2012). Children from low SES develop communication and academic skills slower than children from higher SES (Hirsh-Pasek et al., 2015; Morgan, Farkas, Hillemeier, & Maczuga, 2009). Early intervention and early education programmes should focus on the communication and future academic potential of children to secure the upliftment of vulnerable populations.

Early childhood development classrooms increasingly include typically developing children and a growing number of children at risk for communication delays (Rezzonico et al., 2015) as the number of stay-at-home parents is decreasing (Phillips & Adams, 2001). Children are spending longer hours in the care of ECD practitioners who, with support from health professionals like SLTs, are well placed to facilitate critical early communication development (HSRC, 2012; Mashburn et al., 2008). Extensive literature shows that early intervention services are most effective when strategies are implemented by children’s primary caregivers (Britto et al., 2017). Coaching children’s primary caregivers rather than providing intervention directly better aligns with the (ICF-CY) guidelines (WHO, 2007) as children receive stimulation more frequently and within their functional daily activities. Therefore, because of increased ECD attendance and the role that ECD practitioners play in children’s lives; SLTs should seek out collaborative partnerships with ECD practitioners.

The need for interprofessional collaboration is further highlighted when considering the limited number of SLTs available to treat individuals requiring services (Mayosi & Benatar, 2014). ECD practitioners and SLTs need to reconsider their roles and rather work together to meet the needs of the young, vulnerable population. Interprofessional collaboration is central to the role of SLTs working in early intervention (ASHA, 2008; South African Speech-Language and Hearing Association, 2017). A suitable approach to respond to the identified needs within the classroom could be responsive communication coaching (Archibald, 2017; Glover et al., 2015; Girolametto, Weitzman & Greenberg, 2006 & Friedman, 2015).

Responsive communication is described as consistent and contingent reactions of communication partners, including practitioners, to children’s verbal and non-verbal communication attempts (Flippin & Watson, 2015). Coaching offers a means of customising information for individuals to implement specific strategies within a particular setting (Milburn, Girolametto, Weitzman, & Greenberg, 2014). Recent research has highlighted the benefits of practitioners’ responsivity coaching for communication development (Glover et al., 2015). Coaching improves practitioners’ use of communication facilitation strategies and assists practitioners to embed responsive communication strategies during interactions with young children (Friedman & Woods, 2015; Girolametto et al., 2006), such as identifying and commenting on children’s interests in order to encourage more conversation turns. Responsive communication coaching is predominately applied within the functional setting of the classroom. Collaboration with educators is one of the roles and responsibilities of SLTs working in school-based settings (ASHA, 2010). Classroom-based intervention is outlined as a favourable and viable approach to meeting the needs of young South African learners (Moonsamy & Kathard, 2015) with and who are at risk for communication disorders. However, research on responsive communication coaching and collaboration between ECD practitioners and SLTs in low- to middle-income countries (LMIC), like South Africa, is lacking.

Currently, there are few programmes that guide ECD practitioner–SLT collaboration. These include *Learning Language and Loving It (LLLI) – The Hanen Program for Early Childhood Educators/Teachers* (Weitzman & Greenberg, 2002), Maximizing Academic Growth by Improving Communication (MAGIC), Language-in-Classroom (LIC) and Vocabulary Enrichment Program (VEP) (Archibald, 2017). Although interprofessional collaboration between ECD practitioners and SLTs is identified as advisable, the optimal approach is not yet known (Cirrin et al., 2010). Therefore, reviews of the existing literature currently do not recommend one programme but recommend reason-based practice to guide service delivery (Archibald, 2017; Cirrin et al., 2010; McGinty & Justice, 2006). Further research into interprofessional collaboration between ECD practitioners and SLTs is necessary.

Subsequent to the implementation of interprofessional-based responsive communication coaching, there was a need to reflect on the perceived benefits of the programme. ECD practitioners are one of the primary caregivers in young children’s lives, and therefore, their perceptions of the coaching programme were reviewed.

## Method

### Aim and research design

This study aimed to reflect on ECD practitioners’ perceptions of responsive communication coaching conducted by student SLTs in underserved South African contexts. An explorative research design was implemented.

### Settings

Students were already involved in service delivery at three ECD centres as part of their clinical training and convenience sampling was thus applied. Sites included a preschool in a low socio-economic suburb, a preschool in a small informal community, characterised by poverty, and a preschool catering for children with developmental and special needs. All three preschools required support from non-governmental organisations.

The children, most of whom were English additional language learners, either had or were at risk of communication delays due to environmental and biological factors including, limited maternal education, poverty, HIV and AIDS and cerebral palsy (Table 1). Children attending the special needs preschool presented with communication impairments associated with developmental conditions including autism spectrum disorder, cerebral palsy and Down syndrome.

**TABLE 1:** Overview of settings.

Characteristics	Across all sites
Socio-economic status	Low to middle income
Number of children per classroom	5–35
Gender	Male and female
Age	3 months–5 years
Medium of instruction at ECD centre	English

ECD, early childhood development.

The number of children per site differed significantly. At the special needs preschool, there were five children in each of the four classrooms. The preschool located in the informal settlement included 35 children in one large classroom. The preschool has subsequently expanded, and there are now more classes available and thus smaller class sizes, averaging at about 10 children per class. The suburban preschool classes contained approximately 15 children each in two classes.

### Participants

Fifteen ECD practitioners that the students interacted with at varying sites provided consent to participate in the study. Three participants were educators at the suburban preschool, seven participants were from the preschool for children with special needs and five participants were from the preschool in the informal settlement. There were two ECD practitioners in each classroom, except for one classroom at the suburban preschool where there was only one practitioner. Participants were all female and aged between 20 and 50 years. Although, English was the medium of language for learning and teaching at all three preschools, it was not the first language for most practitioners, which is a common finding in South Africa (Sibomana, 2017). This diversity is a characteristic of South Africa where the majority of the population are multilingual (Samuels et al., 2012).

**FIGURE 1:**
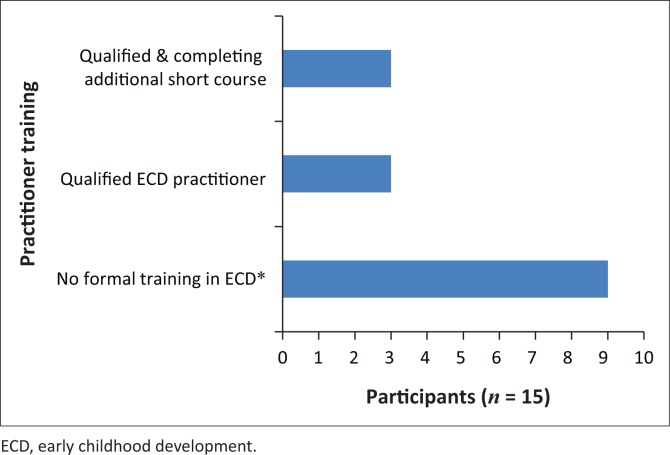
Level of practitioner training across settings.

The educational level and role of the practitioners differed substantially between sites. The range in levels of education, and the fact that most participants (*n* = 9) were not qualified, may be because of previous legislation in South Africa that did not require ECD centres to employ qualified practitioners (HSRC, 2012). Of the nine ECD practitioners with no training (Figure 1), four participants were from the special needs preschool and five were from the preschool in the informal community. However, three of the six practitioners with formal ECD qualifications were completing additional training through tertiary institutions. Two of these practitioners were from the suburban preschool and one was from the preschool for children with special needs. None of the practitioners received responsive communication coaching prior to the sessions with the student SLTs.

### Procedure

The principals and ECD practitioners at all three sites provided consent to participate. The 16-week coaching programme ran from March to August 2016. The student SLTs clinical rotations lasted 6 weeks with four to five senior students in each block and one supervisor per site. Students received seminars regarding interprofessional collaboration and responsive communication coaching. Structured handover occurred between student rotations to ensure treatment fidelity.

At the beginning of the academic year, practitioners completed a checklist identifying their needs, adapted from the Teacher Input Checklist (Tennessee Department of Education, 2009). The checklist was originally designed to help determine how language problems affect educational performance. Additionally, data were collected by students over two sessions through unstructured classroom observations of spontaneous interactions between practitioners and children during classroom routines such as group time, arts and crafts and snack time. Coaching was presented to 15 practitioners via 16 sessions conducted by student SLTs under the guidance of their supervisors. The student SLTs, ECD practitioners and children were divided into small groups in the classroom. The duration (45 minutes) of coaching sessions remained constant; however, the regularity of the site visits differed between settings. The preschool for children with disabilities was visited most frequently, with two coaching sessions provided per week. The preschool in the suburban setting received one session a week, while the site in the informal settlement only received coaching once every second week.

Responsive communication strategies were selected from LLLI (Weitzman & Greenberg, 2002) based on the needs identified by the checklist and classroom observations. This programme was selected as it is most widely used in literature (Bouchard et al., 2010; Cabell et al., 2011; Eadie, Tayler, & Stark, 2017; Girolametto et al., 2006; Glover et al., 2015). LLLI does not require specific classroom materials and can be easily incorporated into any existing preschool curriculum (Cabell et al., 2011). The programme focuses on the promotion of responsive language and overall communication development naturally throughout daily activities and routines in the classroom setting (Weitzman & Greenberg, 2002), such as feeding times, group time and craft activities. Increased conversational turns have been shown to activate language-related areas in the brain, which results in improved language abilities. Reciprocal communication experiences have a greater impact on language development than the quantity of words children are exposed to by their caregivers (Romeo et al., 2018).

Reciprocal communication coaching sessions incorporated components of adult learning such as explaining the relevance of strategies (Friedman, Woods, & Salisbury, 2012), offering descriptions and demonstrations of strategy use, guiding the practitioner through practice, and offering feedback and opportunities for reflections (Dunst & Trivette, 2009). Practitioners were coached to provide responsive language input on children’s topics of interest using child-oriented strategies including ‘observe, wait & listen’ (OWL), ‘face-to-face’ and ‘join in and play’ (Weitzman & Greenberg, 2002). Topics included communication and language development, fostering interaction between children and ECD practitioners, and responsive communication strategies to promote active engagement of children in the communication interaction.

Coaching sessions began with a review of previous strategies and practitioners’ application thereof to classroom settings. Thereafter, the next strategies were discussed and jointly implemented by students and practitioners during classroom routines. As outlined in Friedman and Woods (2015), student SLTs then gave practitioners feedback or suggestions on how to use a strategy (e.g. ‘When he takes the block out of the container, you can copy him and say ‘out’. The meaning of what you say is then very clear to him with the action accompanied by the word’.) or about the child’s response (‘When you paused before giving him another block, he looked at you and made a sound. That gave you a turn and gave him a chance to respond to you’.). Practitioners were encouraged to form an action plan for the incorporation of strategies into the classroom (Friedman & Woods, 2015; Milburn et al., 2014). Supervisors were available during the sessions to assist both SLT students and ECD practitioners.

After 16 weeks of coaching, practitioners completed a self-constructed survey regarding their skill development and experiences of the coaching programme. The survey comprised of four open-ended questions and a Likert scale, ranging from one (not helpful) to five (very helpful), rating the benefit they experienced. The survey evaluated perceived changes in communication and interaction abilities of practitioners, the quality of coaching, overall experience of the coaching process and future needs. Both quantitative and qualitative data were gathered from the survey. Descriptive statistics and thematic content analysis were used to qualitatively analyse the data obtained from the checklist, classroom observations and the survey.

## Ethical consideration

Ethical clearance was obtained from the Research Committee of the Department of Speech-Language Pathology and Audiology, University of Pretoria.

## Results and discussion

In the initial checklist, practitioners identified class size, practitioner–child interaction and diverse needs within the classroom as the main challenges to successful teaching and learning. From a clinical perspective, the student SLTs reported that the unstructured classroom observations of spontaneous interactions revealed a traditional lecture style (Schwerdt & Wuppermann, 2011) of teaching with limited responsive interaction between the practitioners and children. Students also observed busy classroom schedules and practitioner frustration. The identified challenges resonate with recent research (Glover et al., 2015).

The custom-designed survey identified that half of the practitioners (50%) found the coaching very helpful, while 37% perceived it as helpful. The remaining practitioners (13%), based at the special needs preschool, perceived coaching as quite helpful. All practitioners thus expressed benefit from the programme. Research indicates that practitioners perceive a range of benefits from coaching programmes (Friedman & Woods, 2015). The participants from the preschool for children with special needs may have only found the coaching ‘quite helpful’ because most of the practitioners at that site were not qualified (*n* = 4). There is some evidence that certain thresholds of knowledge need to be in place prior to the implementation of coaching programmes (Eadie et al., 2017).

The fact that most of the practitioners (*n* = 14) worked in pairs in their classrooms may have helped them implement the targeted strategies. However, the one practitioner that had a preschool class alone reported that she was excited to ask her colleagues to support her when implementing the strategies with the children. ECD practitioners benefit from the support of other practitioners when applying the strategies covered during responsive communication coaching programmes (Eadie et al., 2017).

Responses from the open-ended survey questions were coded thematically. Three main themes were identified including increased interaction, improved communication abilities and helpful strategies. These themes were contrasted against the initial challenges identified during the needs analysis checklist completed by the ECD practitioners and the unstructured classroom observations by students (Table 2). Survey results indicated that ECD practitioners felt the coaching facilitated reciprocal interaction. Facilitating more effective communication between children and their primary caregivers leads to language development (Romeo et al., 2018) through a more functional ICF-CY (WHO, 2007) approach. The themes are further explained below.

**TABLE 2:** Initial challenges and coaching programme outcomes.

Identified outcomes	Initial challenges						
	Class size	Practitioner–child interaction	Learners’ diverse needs	Limited responsive interaction	Busy classroom schedules	Practitioner frustration	Additional language requirements
Increased interaction	-	√	-	√	-	-	√
Improved communication	√	-	-	-	-	√	√
Helpful Strategies	√	√	√	√	√	√	√

### Increased interaction

Practitioners (60%) reported improved shared attention during interaction. Twenty-five per cent of practitioners perceived improvements in children’s understanding of classroom routines, and 25% felt that they could facilitate improved communication through play. These developments work towards alleviating the limited responsive interaction identified during classroom observations. Classroom-based coaching thus assists ECD practitioners in enhancing children’s communication within daily routines (Friedman & Woods, 2015) as recommended by ICF-CY (WHO, 2007).

### Children’s improved communication

Half of the practitioners perceived improvements in their ability to understand children’s communication attempts. Twenty per cent of practitioners noted improved anticipation of routines, and 20% reported improved communication during daily routines such as mealtimes. These developments were perceived to reduce the stress of large classroom sizes and busy schedules across the sites. Over time, these changes could contribute to improved overall communication within classrooms. Thus, facilitating the development of communication as practitioners engage children more often in responsive, language-rich interactions (Burchinal, Roberts, Zeisel, Hennon, & Hooper, 2006; Vernon-Feagans, Bratsch-Hines, & Family Life Project Key Investigators, 2013).

### Helpful strategies to initiate conversations

The practitioners reported using more responsive communication strategies after the coaching programme. The most useful strategies were identified as ‘observe, wait and listen (OWL)’ (67%), using visual supports to augment verbal communication (33%), providing choices (30%), and using gestures (38%) and face-to-face communication (65%) (Weitzman & Greenberg, 2002). These strategies can promote children’s participation in communication exchanges and increase practitioners’ abilities to respond to children’s diverse communication needs. Conversations centred on children’s interests are processed more easily thereby freeing up cognitive resources for learning (Girolametto et al., 2006).

### Future coaching needs

Practitioners expressed various needs for further coaching including skills to facilitate play and peer-interaction, and to extend the duration of communication exchanges. Results highlight practitioners’ increased awareness of their role in nurturing children’s functional communication abilities. Practitioners at the special needs preschool requested additional training in augmentative and alternative communication strategies. This indicates that the coaching programme needs to be tailored for the diverse populations at each site. Consistent with previous research, results from this study suggest that practitioners have a need for context-based practical strategies for use in the classroom (Glover et al., 2015).

Coaching requires a paradigm shift, from traditional approaches to responsive interaction, by all parties involved and time is needed to adjust to new roles and perspectives (Rezzonico et al., 2015). Continued coaching is required and will be provided, to aid generalisation of strategies to all classroom activities. Generalisation and transdisciplinary teamwork are central to the success of coaching programmes (Friedman & Woods, 2015; Glover et al., 2015). A positive outcome from the study was that role extension, enrichment and expansion were achieved, although not the focus of the coaching programme. Role exchange and release, therefore, require increased focus in future coaching programmes.

### Limitations and suggestions for future research

The implementation of the coaching programme may have been disrupted by student rotations and the variation in frequency of coaching sessions across sites. The number of children in the classrooms at different sites also varied. It should be recognised that it can be extremely challenging to implement new approaches in a large class of 35 children or in a small class of 5 children, where the children have complex developmental challenges and needs. The size of the classrooms may have influenced the application of the responsive communication coaching programme.

The study did not evaluate the outcomes of the coaching programme in relation to practitioners’ respective levels of experience, and a control group of practitioners that did not receive coaching was not included. It would have been insightful to assess children’s communication abilities pre- and post-intervention. However, there was a multitude of potential contributing factors such as additional private, individual SLT. The maintenance of strategies beyond immediate coaching was not formally monitored but collaborative service delivery continued. Additionally, a future consideration is to compare the effect of coaching with in-service workshops to classroom coaching alone. Preliminary results of this study indicate that further research on responsive communication coaching for ECD in LMIC practitioners is warranted.

## Conclusion

ECD practitioners reported that in-classroom coaching sessions led to an increased use of responsive communication strategies and improved interaction and communication with children. Upon reflection of the newly implemented approach, the clinical supervisors were encouraged to continue implementing responsive communication coaching sessions during student’s preschool-based clinical visits. Growing recognition of the link between early language abilities and later academic success emphasises the need for ICF-CY responsive (WHO, 2007) interprofessional collaboration between SLTs and ECD practitioners (Rezzonico et al., 2015). Speech-language therapists and educators present with diverse yet complimentary skills and knowledge sets, which could be beneficial for collaboration and improved ECD (Archibald, 2017). Speech-language therapists need to respond by supporting ECD practitioners in novel ways. The exploratory findings suggest that ECD practitioners benefit from SLT student-led responsive communication coaching sessions.
